# Prognostic Significance of C-Reactive Protein Polymorphism and *KRAS/BRAF* in Synchronous Liver Metastasis from Colorectal Cancer

**DOI:** 10.1371/journal.pone.0065117

**Published:** 2013-06-03

**Authors:** Chi-Jung Huang, Hao-Wei Teng, Chih-Cheng Chien, Jen-Kou Lin, Shung-Haur Yang

**Affiliations:** 1 Department of Medical Research, Cathay General Hospital, Taipei, Taiwan; 2 School of Medicine, Fu Jen Catholic University, New Taipei, Taiwan; 3 Department of Biochemistry, National Defense Medical Center, Taipei, Taiwan; 4 Division of Hematology and Oncology, Department of Medicine, Taipei Veterans General Hospital, Taipei, Taiwan; 5 Institute of Clinical Medicine, National Yang-Ming University, Taipei, Taiwan; 6 School of Medicine, National Yang-Ming University, Taipei, Taiwan; 7 Department of Anesthesiology, Sijhih Cathay General Hospital, New Taipei, Taiwan; 8 Department of Surgery, Taipei-Veterans General Hospital, Taipei, Taiwan; University of Oslo, Norway

## Abstract

**Background:**

The liver is the most common target organ in the metastasis of colorectal cancer (CRC). Synchronous liver metastases may confer a poorer prognosis than metachronous metastases, and genetic alterations and an inflammatory response have also been associated with a poor prognosis in cases of a liver metastasis arising from CRC. However, few studies have examined the relationship between *KRAS* mutations and inflammatory status in CRC, especially with respect to liver metastases.

**Methods:**

The effect of the activated mitogen-activated protein kinase pathway and another protein involved in inflammation, C-reactive protein, in liver metastases were examined. We aimed to determine the impact of the *CRP*-specific single nucleotide polymorphism (SNP) rs7553007 in liver metastasis on the CRC-specific survival (CSS) of patients after colorectal liver metastasectomy.

**Results:**

We found no significant differences in genotype distributions and allele frequencies at the *CRP* SNP rs7553007 between CRC patients with liver metastasis and the control group. CSS rates were low in the subgroup of patients with synchronous metastasis with the A-allele (A/A and A/G) at rs7553007 or mutated *KRAS*/*BRAF* in liver metastatic specimens. Furthermore, the *CRP* SNP rs7553007 (hazard ratio [HR] = 1.101; 95% confidence interval [CI] = 1.011–1.200; *P* = 0.027) and *KRAS/BRAF* mutations (HR = 2.377; 95% CI = 1.293–4.368; *P* = 0.005) remained predictive for the CSS of CRC patients with synchronous liver metastasis in multivariate analysis.

**Conclusions:**

Both the *CRP* SNP rs7553007 and *KRAS/BRAF* mutations were independent prognostic factors for CRC patients with synchronous liver metastasis.

## Introduction

Metastasis comprises a complex series of steps in which cancer cells leave the original tumor site and migrate to a distant organ. Different gene sets with altered expressions may determine different organ targets, along with the metastatic microenvironment [1]. In colorectal cancer (CRC), the liver is the most common target organ of metastasis [2], [3]. Clinically, approximately 25% of CRC patients exhibit synchronous liver metastases at the time of diagnosis, and other patients with advanced disease develop liver metastases within 3 years of treatment [3], [4]. Synchronous metastases, however, may confer a poorer prognosis than metachronous metastases [4]. Recently, genetic heterogeneity were found to be associated with the progression of liver metastasis [1], [5], and the prognosis of liver metastatic CRC was also found to be related to these genetic alterations [6], [7].

The activation of the mitogen-activated protein (MAP) kinase signaling pathway, especially via mutations of *v-Ki-ras2 Kirsten rat sarcoma viral oncogene homolog* (*KRAS*) and *v-Raf murine sarcoma viral oncogene homolog B1* (*BRAF*), is a key step in the development of CRC [8]. The *BRAF* mutation has been shown to be an independent prognostic biomarker for colorectal liver metastasectomy [6], and *KRAS* mutations acquired during the progression of metastatic CRC also have implications for therapy and prognosis [9]. This signaling pathway is also involved in inflammatory processes [10]. C-reactive protein (CRP) is a component of the inflammatory pathway that is dramatically elevated during inflammatory processes and is a useful biomarker for the prognosis of CRC patients [11], [12]. The clinical significance of this protein was also explored with respect to certain polymorphisms of the *CRP* genetic locus [13]–[15]. Several *CRP* single nucleotide polymorphisms (SNPs) were found to be associated with the risk of developing CRC and the survival of patients with this malignancy [16]. However, whether CRP is merely a marker of underlying inflammation or is causally associated with liver metastatic CRC remains uncertain. Thus, studies on the role of inflammation-related modulators in CRC invasion and metastasis are still needed.

Here, we focused on the role of the activated MAP kinase pathway and the inflammation-related protein CRP in liver metastases of CRC patients. The target *CRP* SNP rs7553007 in these metastatic patients was analyzed because of its strong association with CRP levels, which was previously reported in coronary heart disease [15]. We have studied the significance of both a specific SNP of *CRP* and mutations in *KRAS*/*BRAF* in liver metastases with respect to the CRC-specific survival (CSS) of patients after colorectal liver metastasectomy.

## Materials and Methods

### Samples and Clinical Data

From January 2000 to January 2010, 228 patients with colorectal liver metastases (142 patients with synchronous disease and 86 patients with metachronous disease) underwent curative-intent hepatic resection at Taipei Veterans General Hospital, Taiwan. Disease stage was assessed according to the American Joint Committee on Cancer staging system, sixth edition. Clinicopathological staging and clinical course were determined by reviewing a computer database containing detailed information. Non-CRC DNA controls from blood samples (from 79 male and 70 female patients; age range, 50–80 years) were acquired from the Taiwan Han Chinese Cell and Genome Bank. The study protocol was reviewed and approved by the Institutional Review Board of The Taipei Veterans General Hospital. All participants provided written informed consent to participate in this study. Clinical information, including age, sex, the size of the metastatic liver tumor, tumor node metastasis (TNM) stage, and clinical follow-up data, was recorded prospectively (Table 1). Abdominal computed tomography was routinely performed to monitor the presence of metastasis. In addition to the scheduled follow-up examination within 6 months, patients were followed-up at 3-month intervals for up to 2 years, then every 6 months for 5 years, and annually thereafter.

**Table 1:** Clinical features and *KRAS*/*BRAF* analyses of CRC patients.

Feactures and *KRAS*/*BRAF*		n
Age (years)^a^		
	≤62	118
	>62	110
Gender		
	male	140
	female	88
Initial AJCC stage		
	I+II+III	86
	IV	142
Liver metastatic tumor (cm)^a^		
	≤3.5	140
	>3.5	88
Local tumor location^b^		
	proximal	69
	distal	84
	rectal	70
Neoadjuvant chemotherapy^c^		
	No	176
	Yes	52
Adjuvant chemotherapyb,c		
	No	33
	Yes	193
*KRAS* analysis^b^		
	wild type	131
	mutant	
	codon 12	64
	codon 13	21
	codon 14	1
*BRAF* analysis^b^		
	wild type	211
	mutant	
	V599E	2
	V600E	4

**NOTE.** The patient number of CRC-caused death was 70 (initial AJCC stage I/II/III, 20 patients; stage IV, 50 patients). The mean follow-up period was 33.7 months, with the range 0.3 to 129.6 months.

a 62 years, mean age for 228 patients (range, 30–87); 3.5 cm, mean size (range, 0.2–17.2).

b Five patients’ data in local tumor location, 2 (one with initial stage III and one with stage IV) in adjuvant chemotherapy, and 11 in *KRAS*/*BRAF* analyses were unavailable.

c 16 patients with initial stages I/II/III and 36 with stage IV received neoadjuvant chemotherapy; 68 patients with initial stages I/II/III and 125 with stage IV received adjuvant chemotherapy.

### Tissue Genomic DNA Acquisition

For the determination of the *CRP* SNP rs7553007, archived DNA samples of our previous study from 228 liver metastatic specimens were acquired [6]. All liver metastatic specimens were dissected by an experienced gastrointestinal cancer pathologist, and genomic DNA was extracted as described previously [6]. The concentration and purity of the extracts were re-assessed using a NanoDrop spectrophotometer (NanoDrop Technologies, DE, USA).

### Evaluation of the *CRP* SNP rs7553007 and *KRAS* and *BRAF* Mutations

The *CRP* SNP rs7553007 was detected using a TaqMan SNP Genotyping Assay (assay ID: C_26627342_10) in a 7300 Real-Time PCR System (Applied Biosystems, USA), according to the manufacturer instructions [17]. However, this was not possible for some samples, and in these cases, the SNP was evaluated by sequencing using the primers listed in Table S1 and a BigDye Terminator (version 3.1) cycle sequencing kit (Applied Biosystems) on an ABI 3100 capillary electrophoresis system (Applied Biosystems). The *KRAS*/*BRAF* sequences of 217 liver metastatic specimens were obtained from the previous report, but data for the other 11 specimens was missing (Table 1) [6].

### Statistical Analyses

The allele and genotype frequencies of the 228 CRC patients and 149 control subjects were compared using the chi-square test. The clinical and prognostic data of the 228 patients with colorectal liver metastases were analyzed. The analysis of CSS measured from the date of surgery to the date of death from CRC was performed using the Kaplan–Meier survival test, and significance was assessed using the log-rank test. Cox regression was used to assess the prognostic value of the risk category in univariate and multivariate analyses. For the latter, a backward stepwise procedure was used to select the variables that were independent prognostic factors. Variables with a corresponding *P* value of >0.2 were removed from the model. Significance was set at *P*<0.05 (SPSS for Windows version 13.0).

## Results

### Relationship between the *CRP* SNP rs7553007 and Survival Time in CRC Patients with Liver Metastases

**Figure 1:**
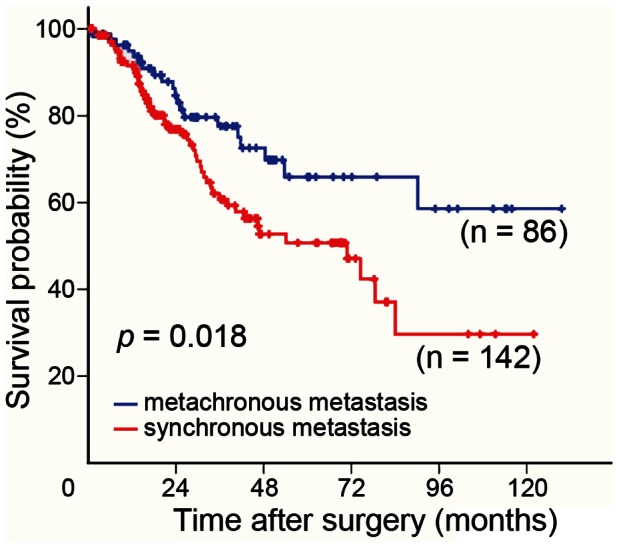
The predicted survival of colorectal cancer patients with liver metastases. Colorectal cancer-specific survival was predicted for patients with synchronous (n = 142) and metachronous (n = 86) metastasis using the Kaplan–Meier survival test. The log-rank test revealed a statistically significant difference between the survival rates.

The CSS rate after colorectal liver metastasectomy was significantly shorter in patients with synchronous metastasis (n = 142) than in patients with metachronous metastases (n = 86) (*P* = 0.018, log-rank test) (Figure 1). The allele and genotype frequencies at rs7553007 were not significantly different between the control subjects and the liver metastatic specimens of patients with both synchronous and metachronous metastasis (Table 2). The frequencies of the A-allele were 57.0% for the 149 control subjects and 55.5% for the 228 liver metastatic patients (53.2% for synchronous metastasis patients and 59.3% for metachronous metastasis patients).

**Table 2:** Genotype frequencies of *CRP* SNP rs7553007 in metastatic CRC patients.

SNP				
	Total cancer	Control	*P* value^a^	*P* value^b^
	(n = 228)	(n = 149)		
AA	70 (30.7%)	47 (31.5%)	0.857	
AG	113 (49.6%)	76 (51.0%)		
GG	45 (19.7%)	26 (17.4%)		
AA *vs*. (AG/GG)			0.579	
(AA/AG) *vs*. GG			0.863	
	Synchronous			
	(n = 142)			
AA	40 (28.2%)		0.605	0.441
AG	71 (50.0%)			
GG	31 (21.8%)			
AA *vs*. (AG/GG)			0.347	0.307
(AA/AG) *vs*. GG			0.530	0.287
	Metachronous			
	(n = 86)			
AA	30 (34.9%)		0.869	
AG	42 (48.8%)			
GG	14 (16.3%)			
AA *vs*. (AG/GG)			0.818	
(AA/AG) *vs*. GG			0.599	

**NOTE.** ^a^Groups were compared for differences in patients (total cancer, synchronous, or metachronous) and control subjects.

b Groups were compared for differences in different (synchronous and metachronous) patients.

**Figure 2:**
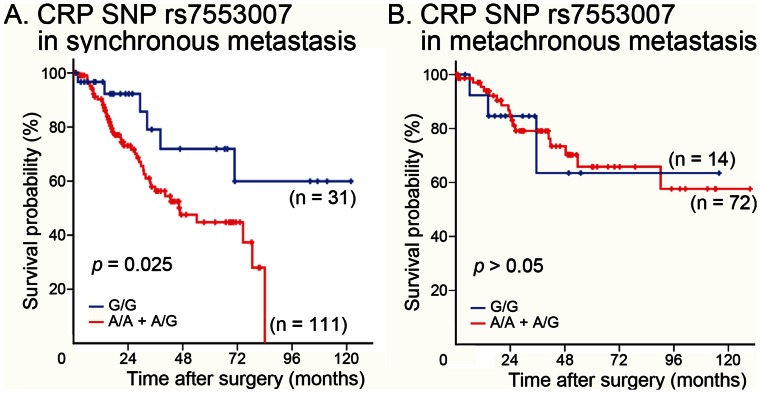
Differences in colorectal cancer-specific survival between patients with different *CRP* single nucleotide polymorphism rs7553007 genotypes. Colorectal cancer (CRC)-specific survival was predicted for patients with synchronous (A) and metachronous (B) metastases. The Kaplan–Meier survival test was used to analyze CRC-specific survival in patients with synchronous (n = 31 for G/G; n = 111 for A/A or A/G) and metachronous (n = 14 for G/G; n = 72 for A/A or A/G) metastases. The log-rank test revealed a statistically significant difference between the survival rates for synchronous metastases.

Among the 142 patients with synchronous metastasis, the subgroup of patients with the A-allele (A/A and A/G) at rs7553007 in liver metastatic specimens (n = 111) had a lower CSS rate than the subgroup of patients without the A-allele (G/G) (n = 31) (Figure 2A). Interestingly, this significant difference in CSS was not noted for the 86 patients with metachronous metastasis (Figure 2B).

### Relationship between *BRAF* and *KRAS* Mutations and Survival Time in CRC Patients with Liver Metastases

**Figure 3:**
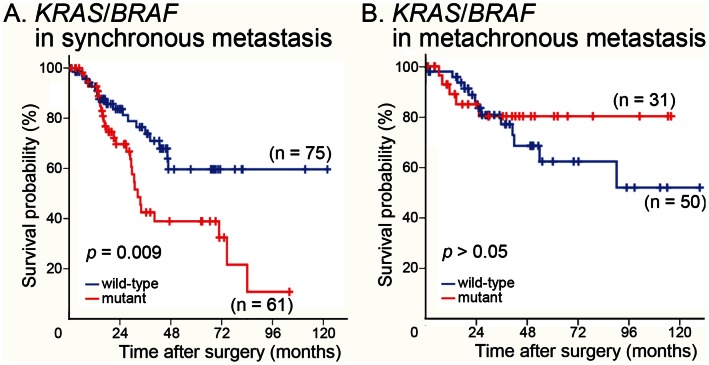
Differences in colorectal cancer-specific survival between patients with different *BRAF* and *KRAS* genotypes. Colorectal cancer (CRC)-specific survival was predicted for patients with synchronous (A) and metachronous (B) metastases. The Kaplan–Meier survival test was used to analyze CRC-specific survival in patients with synchronous (n = 75 for wild-type *KRAS*/*BRAF*; n = 61 for mutants) and metachronous (n = 50 for wild-type *KRAS*/*BRAF*; n = 31 for mutants) metastases. The log-rank test revealed a statistically significant difference between the survival rates for synchronous metastases.

We studied the genetic alterations in the collected liver metastatic specimens. Data on mutated *BRAF* and *KRAS* in liver metastatic specimens from 217 of our patients (136 with synchronous and 81 with metachronous liver metastases) were available from our previous report [6]. The most important finding was that patients with synchronous liver metastasis that had a *BRAF* or *KRAS* mutation had a significantly lower CSS than those with wild-type *BRAF* and *KRAS* (Figure 3A). Conversely, the mutational status of *BRAF* and *KRAS* had no significant association with prognosis in patients with metachronous liver metastasis (Figure 3B).

### Cox Univariate and Multivariate Analyses of CSS in CRC Patients with Synchronous Liver Metastasis

Univariate Cox regression analysis (Table 3) revealed that the *CRP* SNP rs7553007 genotype (hazard ratio [HR] = 1.092; 95% confidence interval [CI] = 1.008–1.183; *P* = 0.030) and *KRAS* or *BRAF* mutations (HR = 2.174; 95% CI = 1.199–3.942; *P* = 0.011) were significantly associated with CSS in CRC patients with synchronous liver metastasis. However, none of the factors listed in Table 3 were predictive of CSS in patients with metachronous metastasis. Significant variables in univariate analysis were included in multivariate analysis (Table 4), and a backward stepwise selection was used for significant predictors. The results showed that the presence of the A-allele at *CRP* SNP rs7553007 (HR = 1.101; 95% CI = 1.011–1.200; *P* = 0.027) and *KRAS/BRAF* mutations (HR = 2.377; 95% CI = 1.293–4.368; *P* = 0.005) were independent prognostic factors in CRC patients with synchronous liver metastasis.

**Table 3:** Univariate Cox regression analyses of cancer-specific survival for patients.

Variables	synchronous metastasis		metachronous metastasis	
	HR (95% CI)^a^	*P* value	HR (95% CI)^a^	*P* value
Age^b^	0.906	0.730	1.617	0.286
young *vs*. old	(0.518–1.586)		(0.669–3.904)	
Gender	0.948	0.852	0.934	0.889
male *vs*. female	(0.543–1.655)		(0.358–2.434)	
Liver metastatic tumor size^b^	1.286	0.391	2.587	0.052
large *vs*. small	(0.724–2.283)		(0.991–6.756)	
Local tumor location	0.835	0.149	1.343	0.149
proximal *vs*. (distal+rectal)	(0.654–1.067)		(0.899–2.005)	
*KRAS*/*BRAF* sequences	2.174	0.011	0.675	0.456
mutant *vs*. wild-type	(1.199–3.942)		(0.240–1.897)	
Genotypes of the *CRP* SNP rs7553007^c^	1.092	0.030	0.991	0.870
(A/A+A/G) *vs*. G/G	(1.008–1.183)		(0.886–1.108)	

**NOTE.** ^a^HR, hazard ratio; CI, confidence interval.

b Using the mean age of patients (62 years for synchronous metastasis and 65 years for metachronous metastasis) and the mean size of tumors (3.6 cm for synchronous metastasis and 3.3 cm for metachronous metastasis) as cut-off values.

c Genotypes for *CRP* SNP rs7553007, non-A-allele carrier (GG), and A-allele carrier (AA/AG).

**Table 4:** Multivariate Cox regression analyses of cancer-specific survival for patients.

Variables	HR (95% CI)^a^	*P* value
*KRAS*/*BRAF* sequences	2.377 (1.293–4.368)	0.005
mutant *vs*. wild-type		
Genotypes of *CRP* SNP rs7553007^b^	1.101 (1.011–1.200)	0.027
(A/A+A/G) *vs*. G/G		
Local tumor location	0.811 (0.620–1.061)	0.126
proximal *vs*. distal+rectal		

**NOTE.** ^a^HR, hazard ratio; CI, confidence interval.

b Genotypes for *CRP* SNP rs7553007, non-A-allele carrier (GG), and A-allele carrier (AA/AG).

## Discussion

As expected, the prognosis of CRC patients with synchronous liver metastasis was generally worse than that of patients with metachronous liver metastasis. The synchronous presence of primary colorectal tumors and liver metastasis might indicate a more disseminated disease status and was associated with a shorter disease-free survival than metachronous metastasis [18]. Ishizuka et al. further reported that the systemic inflammatory response reflected by preoperative serum CRP correlates with the disease outcome in these liver metastatic patients [19]. Thus we assume that synchronous liver metastasis will correlate with stronger systemic inflammation status.

To improve therapeutic efficacy, the management of CRC patients with synchronous or metachronous liver metastases is individualized according to the needs of each patient [20]. It is therefore important to understand the factors that influence outcome after resection of liver metastases [21].

Both *CRP* SNPs and CRP levels have been widely studied in coronary heart disease (CHD) [22], [23]. Similarly, CRP has been shown to be associated with the risk of colorectal neoplasia [16], [24]. However, findings regarding the relationship of *CRP* genetic variants and CRP levels with disease risk have been contradictory in recent reports [15], [23]. Elliott et al. found that the presence of the A-allele at *CRP* SNP rs7553007 was associated with low CRP levels but not with CHD risk [15]. In the present study, we found that CRC patients with synchronous liver metastasis harboring the A-allele had a significantly poor prognosis. This finding is somewhat different from the expected effect of the A-allele, based on the study by Elliott et al. These conflicting findings may be the result of differences both in the ethnicity of the patients and the type of disease. Most of the participants in Elliott’s study were European. As listed in Table S2 (data from the NCBI SNP database: www.ncbi.nlm.nih.gov/projects/SNP/snp_ref.cgi?rs=7553007), the A-allele is less common in European (33.6%) and African populations (19.9%) than in Asian populations (over 50%), and certain subpopulations have even higher frequencies, including the Han Chinese population in Beijing (55.6%) and the Japanese population in Tokyo (73.3%). The different results may also be explained by the complex inflammatory responses. Whether CRP is merely a marker of underlying inflammation or is causally associated with liver metastatic CRC remains uncertain. An inflammatory response associated with *KRAS*/*BRAF* mutations has been reported in many human cancers, including CRC [25]–[28]. In the MAP kinase pathway, *KRAS*/*BRAF* mutations occur in a mutually exclusive manner [29]–[31]. A previous study showed that http://onlinelibrary.wiley.com/doi/10.1002/ijc.25042/full - bib4#bib4*BRAF* mutations were associated with the absence of peritumoral lymphocytic inflammation and a poor prognosis [28].

In agreement with the conclusions of previous studies, we propose that some inflammatory responses create an environment that fosters tumor cell growth, invasion, and dissemination [32]. Thus, an inflammatory response caused by a *KRAS* mutation and certain *CRP* genotypes may aggravate colorectal liver metastases. In this study, we showed that the presence of the *CRP* SNP rs7553007 (A-allele) in liver metastatic tumors was a significant risk factor for a poor prognosis in CRC patients with synchronous liver metastases, but not in patients with metachronous liver metastases. The survival difference between patients with synchronous and metachronous liver metastases has also been reported in a number of other studies. [4], [33], [34].

As discussed by Nordlinger et al., perioperative chemotherapy plus liver resection had been shown to be better than liver resection alone [35]. In our study, neither neoadjuvant nor adjuvant chemotherapy had effect on the survival of patients with synchronous metastasis. This might be caused by the heterogenous regimens of chemotherapy plus target therapy adopted for our patients. Only patients with metachronous metastasis had significantly survival benefit of receiving adjuvant chemotherapy. It highlights not only the apparent benefit of chemotherapy for metachronous group, but also the different natures between of these two groups.

To our knowledge, this is the first study to show an association between the specific *CRP* SNP rs7553007 and the prognosis of CRC. CRP is a component of the inflammatory pathway that is dramatically elevated during inflammation, and a high CRP level has been shown to be associated with poor survival [11], [36]. Although we did not have data on CRP levels for the patients enrolled in this study, the *CRP* SNP rs7553007 was of clinical significance. According to genotyping results, the proportions of the A-allele of the *CRP* SNP rs7553007 are similar in control subjects and CRC patients with liver metastasis, as is the case in other Asian series [15].

In conclusion, our results suggest that both the presence of the A-allele of the *CRP* SNP rs7553007 and *KRAS*/*BRAF* mutations are significant prognostic factors for CRC patients with synchronous liver metastasis. The nature of the inflammatory responses affected by the *CRP* SNP and specific *KRAS*/*BRAF* mutations should be studied further.

## Supporting Information

Table S1 **Primer sequences to determine CRP SNP rs7553007.**
(DOC)

Table S2 **Allele frequencies of CRP SNP rs7553007 in different population.**
(DOC)
