# Supplementary material for: Prognostic Significance of C-Reactive Protein Polymorphism and KRAS/BRAF in Synchronous Liver Metastasis from Colorectal Cancer
Source: PLoS One. 2013 Jun 3;8(6):e65117. doi: 10.1371/journal.pone.0065117 (PMC3670930; doi:10.1371/journal.pone.0065117)
Supplement: Table S1 — Primer sequences to determine CRP SNP rs7553007. (DOC) [file pone.0065117.s001.doc]

| **Table S1.** Primer sequences to determine *CRP* SNP rs7553007 | |
| --- | --- |
| **Features** |  |
| Chromosome number | 1 |
| Accession number | AF442818 |
| Amplicon size (bp) | 481 |
| Primer sequences | Forward: 5’-GAGTGGGAGAAGGGTCATCA-3’ |
|  | Reverse: 5’-TACCCATGGCCAAGCTACTC-3’ |
| **NOTE.** The annealing temperature to amplify target fragments is 65°C. | |
