# Supplementary material for: Prognostic Significance of C-Reactive Protein Polymorphism and KRAS/BRAF in Synchronous Liver Metastasis from Colorectal Cancer
Source: PLoS One. 2013 Jun 3;8(6):e65117. doi: 10.1371/journal.pone.0065117 (PMC3670930; doi:10.1371/journal.pone.0065117)
Supplement: Table S2 — Allele frequencies of CRP SNP rs7553007 in different population. (DOC) [file pone.0065117.s002.doc]

| **Table S2.** Allele frequencies of *CRP* SNP rs7553007 in different population | | | | |
| --- | --- | --- | --- | --- |
| **Population** | **Individual Group** | **Chromosome Sample Count** | **Allele A** | **Allele G** |
| Chinese | Asian |  |  |  |
| Beijing |  | 90 | 55.6% | 44.4% |
| Shanghai |  | 160 | 57.5% | 42.5% |
| Metropolitan Denver |  | 170 | 50.6% | 49.4% |
| Japanese | Asian | 172 | 70.3% | 29.7% |
| Utah residents | European | 226 | 33.6% | 66.4% |
| Yoruba | African | 226 | 19.5% | 80.5% |
| **NOTE.** Data were acquired from the NCBI SNP database: www.ncbi.nlm.nih.gov/projects/SNP/snp_ref.cgi?rs= 7553007. | | | | |
